# Medico-Legal Section

**Published:** 1911-11-11

**Authors:** Walter Asten

**Affiliations:** Barrister-at-Law


### MEDICO-LEGAL SECTION.

### Traumatic Neurasthenia.

By WALTER ASTEN, L.R.C.P., L.R.C.S., Barrister-at-Law.*

Although much has been said and written on traumatic neurasthenia, considerably more remains to be said before the last word can by any means be declared. It was quite natural to anticipate that this diagnosis would become much more prominent after the passing of the Workmen's Compensation Act, and while it may be stated that only a small number of these cases are the subject of damages *inside* the courts, it will not be contested that *outside* the courts a large number give rise to trouble and annoyance to the parties concerned.

The term traumatic neurasthenia has come to be used very loosely and irregularly, judges, doctors, and lawyers being all more or less guilty. There is very great difficulty in enunciating precise and limited definitions of the various functional neuroses as the symptoms presented by each run into the others, but experience of claims arising under the Workmen's Compensation Act emphasises the necessity of greater exactness and classification where possible.

In the case of Eaves *v.* Blaenclydach Colliery Co., Ltd. (2 K.B., 1909, p. 73) the county court judge considered the evidence proved that the applicant was suffering from traumatic neurasthenia and anæsthesia of the leg as a consequence of the accident, that traumatic neurasthenia was a real complaint, and that it had affected the applicant's mental condition and destroyed his will power. In the court of appeal on this case the Master of the Rolls said: "The effects of an accident are at least twofold; they may be merely muscular effects, they almost always must include muscular effects, and they may also be and very frequently are effects which you may call *mental*, or *nervous*, or *hysterical*, whichever is the proper word to use in respect of them. The effects of this second class as a rule arise as directly from the accident which the workman suffered as the muscular effects do, and it seems to me entirely a fallacy to say that a man's right to compensation ceases when the muscular mischief is ended, but the nervous effects still remain."

This is a very important pronouncement, but clearly the Master of the Rolls and the county court judge intend the term "traumatic neurasthenia" to be comprehensive and to cover the whole multitude of nervous affections of functional order.

Some reasonably approximate differentiation ought to be secured between the three states, traumatic neurasthenia, traumatic hysteria, and purposeful malingering. It would be well to get back to the original idea conveyed by the term traumatic neurasthenia. It was ascribed to the train of symptoms set up after any sudden concussion of the brain or spinal cord in which there were no evidences of external injury; railway accidents being said to be the most common cause. Briefly, there is the history of accident with consequent shock and mental excitement, followed at varying intervals of time by subjective symptoms, such as pain and tenderness over certain regions of the spine, inability to suffer any serious physical or mental effort, palpitation, headache, despondency, loss of memory, and in bad cases the development of certain phobias.

Now this is a genuine succession of events which, to the patient at any rate, is often a very serious burden. But it should be remembered that all these symptoms may arise in one who has suffered but a very trivial injury, or even as a result of mere fright in witnessing some harrowing scene. Again it is well known there are certain predisposing influences in the individual, such as hereditary tendency.

The late Judge Emden said in January of this year that the number of workmen suffering from neurasthenia was extraordinary, and he felt sure that none of these workmen knew what the malady was until the Workmen's Compensation Act was passed. There can be no doubt that in some instances the malingerer does simulate neurasthenic symptoms extraordinarily cleverly. It is desirable here to enter a protest against the statement sometimes made to the effect that the general practitioner is slow to detect malingering. He has the advantage of examining the workman in his own locality and being probably acquainted with his general character and life-history, and hence uncommonly well fitted to judge between a supposed case of malingering and some hereditary predisposition to nerve weakness.

There are cases in which it may be quite obvious that there is no malingering and no evidence of serious illness, yet the man is physically unfit for work. It is best, however, to limit the term "hysteria" to those emotional disturbances, morbid sentiments, or psychic neuroses induced solely by the patient's imagination, hypersensibility, or self-persuasion in which no history of shock or concussion involving the central nervous system is obtained. It would be an advantage to retain the old idea of hysteria with which the laity are familiar, viz. that it chiefly affects the female sex, and that the patient can prevent the symptoms if so minded.

The previous condition of health and predisposition to nervous disease ought to be an important factor in determining an employer's liability. Naturally enough, the wise employer refuses the risk of employing men whom he knows to be unsound, although perhaps capable, workmen, since he is not allowed to contract out of his liability on their behalf. Provision for contracting out with proper safeguards is most necessary, unless it is desired to place a premium upon malingering. Amendments to the Act along these lines would have the effect of making it more uniform in practice and less complex in administration.

* From a paper read before the Medico-Legal Society, October 17, 1911.

